# Clinical and genetic features of Spanish patients with Mevalonate kinase deficiency

**DOI:** 10.1186/1546-0096-13-S1-P36

**Published:** 2015-09-28

**Authors:** E Ruiz-Ortiz, E Gonzalez-Roca, A Mensa-Vilaro, J Rius, S Plaza, C Anton, I Calvo, C Modesto, J Anton, C Arnal, C Alvarez, J Alvarez-Coca, E Becerra, N Bilbao, M Camacho, J Crespo, C de Diego, LF Diez-Garcia, L Espinosa, D Garcia-Escriva, F de Gracia, MI Gonzalez, E Iglesias, S Izquierdo, B Lastra, P Llobet, B Lopez, V Lopez-Gonzalez, R Martinez, MA Martin-Mateos, R Merino, L Ortega, ME Peiro, I Perez de Soto, C Perez-Mendez, V Rodriguez-Valverde, A Ribes, A Ruiz, B Sanchez, JL Santos, B Sevilla, J Sotoca, J Vilas, A Villoria, J Yagüe, JI Arostegui

**Affiliations:** 1 Hospital Clinic, Barcelona, Spain; 2 Hospital La Fe, Valencia, Spain; 3 Hospital Vall Hebron, Barcelona, Spain; 4 Hospital Sant Joan De Deu, Barcelona, Spain; 5 Hospital Marques De Valdecilla, Santander, Spain; 6 Hospital Del Niño Jesus, Madrid, Spain; 7 Hospital Universitario De Torrevieja, Alicante, Spain; 8 Hospital Virgen Del Rocío, Sevilla, Spain; 9 Hospital Virgen De La Salud, Toledo, Spain; 10 Complejo Hospitalario Torrecárdenas, Almeria, Spain; 11 Hospital La Paz, Madrid, Spain; 12 Hospital General De Valencia, Valencia, Spain; 13 Hospital Virgen De La Luz, Cuenca, Spain; 14 Hospital Miguel Servet, Zaragoza, Spain; 15 Hospital Central De Asturias, Oviedo, Spain; 16 Hospital De Granollers, Barcelona, Spain; 17 Hospital Universitario Virgen De La Arrixaca, Murcia, Spain; 18 Complexo Hospitalario Universitario De Ourense, Ourense, Spain; 19 Hospital Universitario Virgen De Las Nieves, Granada, Spain; 20 Hospital De Cabueñes, Gijon, Spain; 21 Hospital Son Espases, Mallorca, Spain; 22 Hospital San Cecilio, Granada, Spain; 23 Complejo Hospitalario Universitario De Albacete, Albacete, Spain; 24 Hospital De Pontevedra, Pontevedra, Spain; 25 Corporació Parc Taulí, Barcelona, Spain

## Introduction

Mevalonate kinase deficiency (MKD) is a recessively-inherited autoinflammatory condition caused by *loss-of-functionMVK* mutations. This gene encodes for the enzyme mevalonate kinase (MVK), which catalyzes a crucial step of the biosynthetic pathway of cholesterol and isoprenoids. The partial deficiency of enzymatic activity causes the Hyper-IgD and periodic fever syndrome (HIDS), whereas it complete deficiency provokes the Mevalonic Aciduria (MA).

## Objectives

The aim of this study was to describe the clinical and genetic features of Spanish patients with MKD diagnosed during the past 15 years.

## Methods

The patients’ data as well as the outcome of the administered treatments were collected from charts reviews. *MVK* analysis was performed by Sanger-based sequencing.

## Results

Forty-one patients from different Spanish hospitals were included. Thirty-eight patients (92.7%) suffered from HIDS and three patients (7.3%) from MA. The MKD diagnosis was established in all of them by the detection of biallelic *MVK* mutations. Eighteen different *MVK* mutations were detected, with the p.[(V377I)] and p.[(I268T)] mutations as the most prevalent, accounting for 54.9% and 26.8% of mutated alleles, respectively. The majority of these mutations (96.4%) were missense mutations. The remainder mutations included premature stop (1.2%), frameshift (1.2%), and splice site mutations (1.2%). In the group of patients with HIDS (n: 38), twelve patients (31.6%) carried homozygous genotypes and twenty-six patients (68.4%) compound heterozygous genotypes. In the group of HIDS patients with homozygous genotypes (n= 12), ten patients (83.3%) carried the p.[(V377I)];[(V377I)] genotype. By contrast, in the group of patients with MA only one patient (33.3%) carried a homozygous genotype (the p.[(I268T)];[(I268T)]).

From a clinical point of view, the median age at the disease onset was 6 months (range 0-408), and the median duration of flares was 4.8 days (range 2-17.5). Mandatory vaccinations were identified as triggering factors for acute episodes in eleven patients (26.8%). The most prevalent manifestations during inflammatory episodes were fever (80.5%), lymphadenopathies (70.7%), abdominal pain (63.4%), diarrhea (58.5%), aphthous ulcers (53.7%) and arthralgia (51.2%). AA amyloidosis was only detected in one patient (2.4%), but had a severe course.

## Conclusion

We herein provide a detailed description of the clinical and genetic features of a Spanish cohort of MKD patients carrying biallelic *MVK* mutations. Most of patients suffered from the mild MKD phenotype, the HIDS syndrome. Two prevalent *MVK* mutations, p.[(V377I)] and p.[(I268T)], were found in our cohort, and fever and lymphadenopathies were the most common features in enrolled patients.

